# MRI-Based Radiomic Model for Preoperative Risk stratification in Stage I Endometrial Cancer

**DOI:** 10.7150/jca.50872

**Published:** 2021-01-01

**Authors:** Jingya Chen, Hailei Gu, Weimin Fan, Yaohui Wang, Shuai Chen, Xiao Chen, Zhongqiu Wang

**Affiliations:** 1 Department of Radiology, Affiliated Hospital of Nanjing University of Chinese Medicine, Jiangsu Province Hospital of Chinese medicine, Nanjing, Jiangsu Province, China.; 2 Department of radiology, Women's Hospital of Nanjing medical University (Nanjing Maternity and Child Health Care Hospital), Nanjing, Jiangsu Province, China.; 3 Department of Clinical Laboratory, Women's Hospital of Nanjing medical University (Nanjing Maternity and Child Health Care Hospital), Nanjing, Jiangsu Province, China.; 4 Department of Pathology, Affiliated Hospital of Nanjing University of Chinese Medicine, Jiangsu Province Hospital of Chinese medicine, Nanjing, Jiangsu Province, China.; 5 Department of Radiology, The Eighth Affiliated Hospital, Sun Yat-sen University, Shenzhen, Guangdong Province, China.

**Keywords:** MRI, radiomic, endometrial cancer, risk, nomogram

## Abstract

**Introduction:** Preoperative risk stratification is crucial for clinical treatment of endometrial cancer (EC). This study aimed to establish a model based on magnetic resonance imaging (MRI) and clinical factors for risk classification of EC.

**Materials and Methods:** A total of 102 patients with pathologically proven Stage I EC were included. Preoperative MRI examinations were performed in all the patients. 720 radiomic features were extracted from T2-weighted images. Least absolute shrinkage and selection operator (LASSO) regression model was performed to reduce irrelevant features. Logistic regression was used to build clinical, radiomic and combined predictive models. A nomogram was developed for clinical application.

**Results:** The radiomic model has a better performance than the model based on clinical and conventional MRI characteristics [AUC of 0.946 (95% CI: 0.882-0.973) vs AUC of 0.756 (95% CI: 0.65, 0.86)]. The combined model consisting of radiomic features and tumor size showed the best predictive performance in the training cohort with AUC of 0.955 in the training and 0.889 in the validation cohorts.

**Conclusions:** MRI-based radiomic model has great potential in prediction of low-risk ECs.

## Introduction

Endometrial cancers (EC) are the most prevalent malignant neoplasms of the female genital system 1. Its incidence is now increasing owing to the advances in medical imaging. About 80% of the ECs are diagnosed in early stage, with 5-year survival rates of over 95% 2. According to the ESMO-ESGO-ESTRO clinical practice guideline for EC 3, the stage I ECs are stratified into three risk stratifications: low risk (Stage IA endometrioid, grade 1-2); intermediate risk (Stage IA endometrioid, grade 3; or Stage IB endometrioid, grade 1-2); high risk (Stage IB endometrioid, grade 3). The prognosis and recommended treatment approach of patients are different due to different risk stratification. The risk classification of stage I EC has been developed to prevent over-treatment or under treatment of EC in clinical practice. It is not recommended for low-risk ECs to perform lymphadenectomy since it may increase complications without overall survival benefits. However, lymphadenectomy may improve the outcome significantly in patients of intermedium-high risk EC 4. So, it is important to find a useful method to assess the risk stratification of stage I EC to guide decision-making precisely.

MRI features have been proved valuable in discriminating different risk groups of EC 5-8. Liu et al. 8 described the ability of apparent diffusion coefficient (ADC) values in assessment of early-stage EC patients based on risk categories. K et al. 9 also evaluated the performance of predicting lymph node involvement of three risk-stratification models in endometrioid EC, the best model gained an area under curve (AUC) of 0.780. Although these clinical and imaging features may be helpful in practice, it is hard to make a reliable differentiation among risk groups.

Radiomics has been applied to clinical-decision systems to improve diagnostic, prognostic, and predictive accuracy in recent years 10-13. Radiomic features offer information about tumor microenvironment that is complementary to other clinical or imaging data. Furthermore, MR imaging-based texture features have been found to be associate with the risk stratifications of EC 12, 14. A recent study reported that MR texture analysis could predict deep myometrial invasion and high-risk group of ECs independently 15. They concluded that texture analysis could serve as imaging biomarkers for preoperative risk assessment. However, these previous results were focusing on predicting the status of lymph node metastasis, which is one of the important factors for EC risk stratification. Yan et al. 12 developed a radiomics nomogram for preoperatively prediction of high-risk EC, and the model achieved good performance. The risk of stage I EC was always underestimated because of insufficient information provided by conventional MRI features, the investigation of MRI based radiomic analysis combined with clinical features in preoperative assessment of risk stratification of stage I EC remains to be elucidated.

The purpose of this study was to investigate the effectiveness of MRI derived radiomic parameters combined with clinical features for the preoperatively evaluation of risk stratification of stage I EC.

## Materials and methods

### Study population

**Figure 1:**
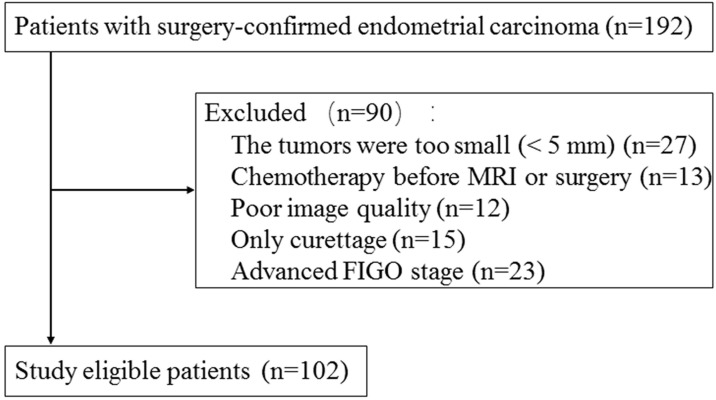
Flowchart of study population selection and exclusion criteria.

This retrospective study was approved by the institutional review board and informed consent was waived. A total of 192 consecutive patients with histologically proven EC between September 2018 and November 2019 in our hospital were included. Pelvic MRI was performed within two weeks of surgery on all and then totally hysterectomy was performed. The exclusion criteria were the following: 1) the largest diameter of lesion < 5 mm (n = 27); 2) patients underwent chemotherapy before pelvic MRI examination or surgery (n = 13); 3) poor image quality (n = 12); 4) only diagnostic curettage was performed (n = 15); 5) advanced stage (n = 23). Finally, this study included 102 patients with an age of 57.8±9.7 years (mean ± SD) (Fig. 1). The ECs consisted of 69 low-risk tumors, 22 intermediate risk tumors, and 11 high-risk tumors. The patients were divided into two groups: low-risk ECs and intermediate-high risk ECs. 49 low-risk tumors and 21 intermediated-high risk tumors were randomly selected as training cohort and the remaining 32 tumors were assigned to validation cohort.

### MRI Imaging

All of the examinations were executed using a 1.5-T system MRI (Magnetom Aera; Siemens Healthcare, Germany) using body phase array coil. The transverse planes covered the entire pelvis. Axial T1-weighted imaging (TR/ TE = 139/4.76 ms; interaction gap = 1 mm; thickness = 5 mm), axial T2WI (TR/TE = 1900/76 ms; interaction gap = 1 mm; thickness = 5 mm), sagittal fat-suppression T2-weighted imaging (TR/TE = 263/4.76 ms; interaction gap = 1 mm; thickness = 5 mm) were obtained. Sagittal diffusion weighted image (DWI) was obtained (TR/TE = 6900/80 ms; interaction gap = 1 mm; thickness = 5 mm) with b-value of 50 and 800 s/mm^2^, ADC maps were automatically generated on the post-processing workstation.

### Clinical and Conventional MRI assessment

The conventional MRI images were analyzed by a genitourinary radiologist with 10 years of experience and by a pelvic radiologist with 8 years of experience. They were blinded to the pathological results. Clinical characteristics and conventional MRI features were concluded to differentiate low-risk ECs from intermediated-high risk ECs. The following features were evaluated: age, menopausal status, tumor size, tumor margin, signal on DWI, T1WI and T2WI signal intensity, and deep myometrial invasion. Size of each tumor was measured on the cross section. The cases with discrepancies were discussed by the two radiologists again to reach an agreement. The association between risk stratifications and clinical and conventional MRI characteristics was evaluated using univariate analyses. The statistically significant (*p* < 0.05) features from the univariate analyses were used to develop the clinical model using multivariate analyses.

### Radiomic analysis

**Figure 2:**
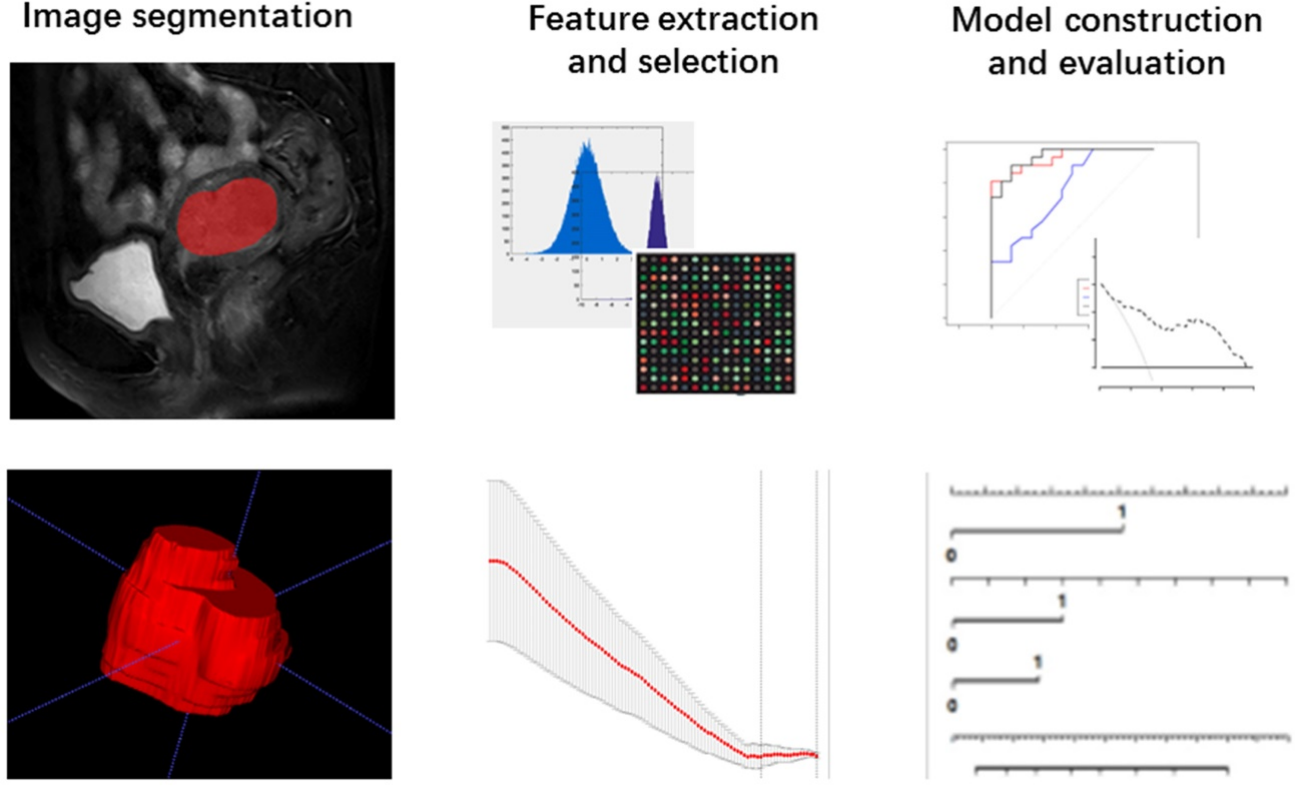
Workflow of the radiomic analysis.

**Figure 3:**
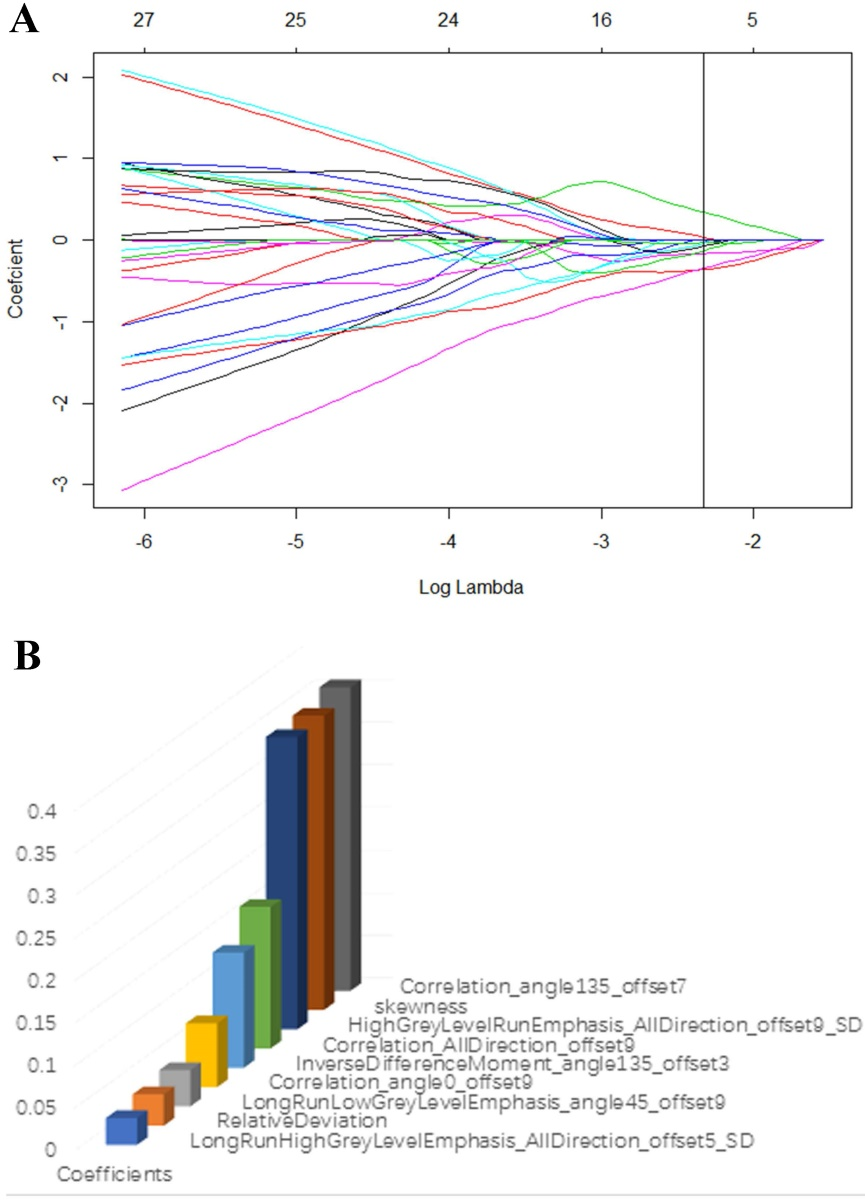
(A) LASSO regression model was used to select radiomic features in the training cohort. (B) Radiomic features that retained for further model establishment.

The T2-weighted were acquired from pictures archiving and communication system (PACS) for Radiomic analysis. The whole volume of interest containing the entire visible tumor was drawn manually using ITK-SNAP software (http://www.itksnap.org). A commercial software package (Artificial Intelligent Kit-A.K, GE Healthcare) was used to extract the radiomic features. Mann-Whitney U tests were firstly adopted to reduce redundant information. Features with *p* < 0.1 were reserved. Then, a least absolute shrinkage and selection operator (LASSO) regression model was performed to reduce irrelevant features in the training cohort (Fig. 3A). The model works by defining the coefficients of irrelevant factors as zero with the regulation parameter. The workflow of image segmentation, radiomic feature extraction, prediction model establishment is described in Fig. 2. Each radiomic feature was weighted by a single coefficient derived from multi-factors linear regression. The radiomics score for every single patient was computed using the linear combination of feature coefficients. A combined model was build using the logistic regression method in the training cohort based on the statistically significant (*p* < 0.05) radiomic signature and the clinical features. The efficacy of the combined model was tested in the validation cohort, a nomogram of a combined clinical and radiomic model was plotted.

### Statistical analysis

The Independent sample t-test was used for normal distribution data. The Mann-Whitney U-test was used for non-normal distribution data. Pearson's chi-squared test was used to test categorical variables of different risk EC groups. The Mann-Whitney U test was used to compare the texture parameters on ADC maps and clinical features between groups. The diagnostic efficiency of each approach was evaluated by receiver operating characteristic (ROC) curve and area under the curve (AUC). The clinic efficacy of each model was evaluated by comparing the AUC of each predictive model using DeLong's test. The decisive curve analysis, integrated discrimination index (IDI), and net reclassification index (NRI) were employed to evaluate the net benefit of predictive model. IBM SPSS 22.0 (IBM corporation, NEW York) for Windows and R software (v. 3.7.0; http://www.r-project.org/) was employed for these analyses. A nomogram was built based on the varibales derived from the combined model for predicting the probability of low-risk ECs. *P* < 0.05 was considered statistically significant.

## Results

### Clinical and Conventional MRI characteristics

The clinical, pathological and conventional MRI characteristics of 102 ECs are available in Table 1 and Table 2. There were no statistical differences between the training and the validation cohort on all of the clinical features. There were 69 low-risk, 22 intermediated-risk, and 11 high-risk ECs based on the histological findings. The mean maximal diameter (standard ± deviation) was 2.68 ± 1.12 cm for the low risk group and 4.08 ± 1.95 cm for the intermediated-high risk group. Intermediated-high risk group were significantly larger than low-risk tumors (*p* < 0.01). The DMI status was statistically significant between these two groups. No statistical differences between the low risk and intermediated-high risk ECs were detected for age, tumor signal intensity on the DWI, T1WI and T2WI images, and tumor margin. Logistic regression analysis showed that size and DMI were factors for the prediction of low-risk ECs. The clinical model of the combined features of tumor size and DMI yielded an AUC of 0.756 (95% CI: 0.65-0.86) with the sensitivity and specificity of 85.5% and 71% in the training cohort, and an AUC of 0.704 (95% CI: 0.40-1) with the sensitivity and specificity of 94.3% and 53% in the validation cohort (Fig. 5).

### Radiomic Features

Seven hundred and twenty radiomic features were extracted from T2-weighted images, consisting of 9 first order features, 42 histogram features, 288 grey level co-occurrence matrix (GLCM) features, 360 run length matrix (RLM) features, and 11 grey level size zoon matrix (GLSZM) features. After sufficient dimension reduction, nine features were selected for radiomic model building. Detailed information of these features is showen in Table 3, Fig. 3B, and Fig. 3C. The radiomic model showed a significant ability in classification risk groups with an AUC of 0.946 (95% CI: 0.882-0.973), with a sensitivity of 81% and specificity of 96.5% in the training cohort and an AUC of 0.815 (95% CI: 0.588-1), with a sensitivity of 76.8% and specificity of 83.3% in the validation cohort (Fig. 5).

### Radiomic Nomogram Construction

Radiomic signature and clinical characteristics extracted in previous steps were analyzed, and a combined model was built using multivariable logistic regression. The predictive performance of this combinated model yielded an AUC of 0.955 (95% CI: 0.899-01) in the training cohort, and an AUC of 0.889 (95% CI: 0.7-0.989) invalidation cohort.

**Figure 4:**
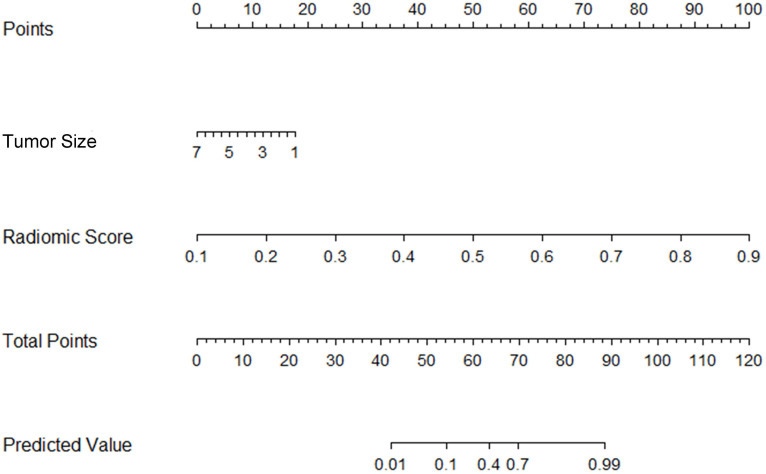
Preoperative nomogram for the combined model. The different value for tumor size and radiomic score corresponds to a point on the line. Total point is calculated by adding all the points up. And the final predicted value was corresponded to the total point, which could be used in clinic conveniently.

**Figure 5:**
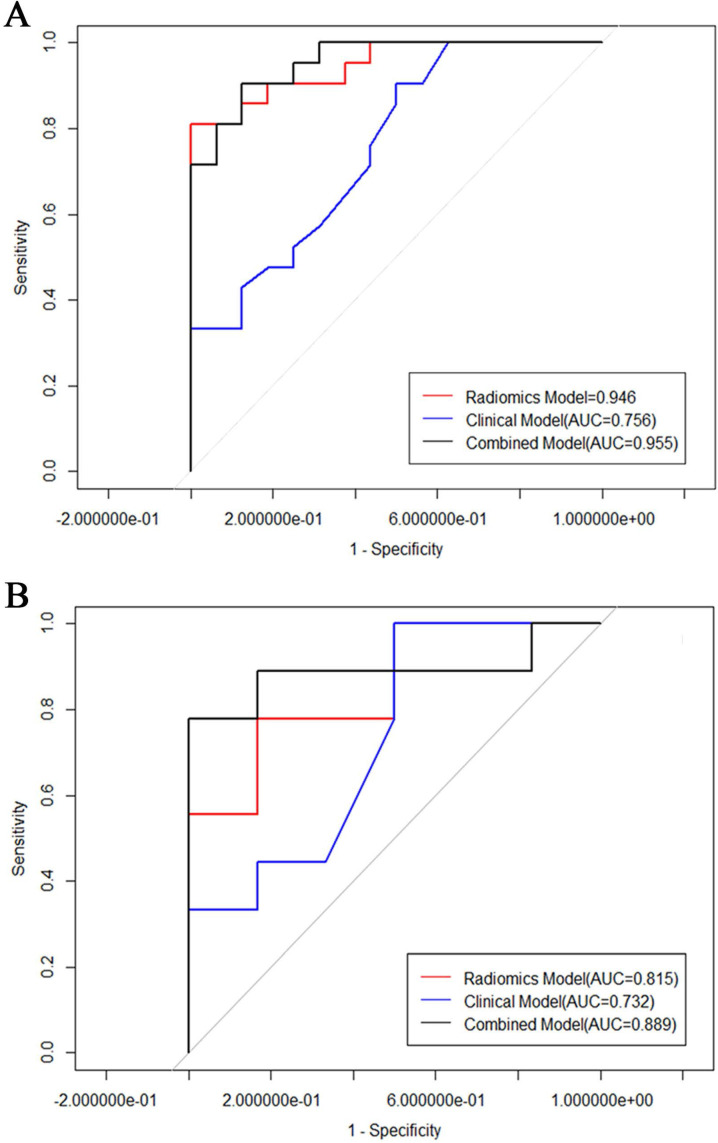
ROC of the models in training cohort (A) and test cohort (B).

**Figure 6:**
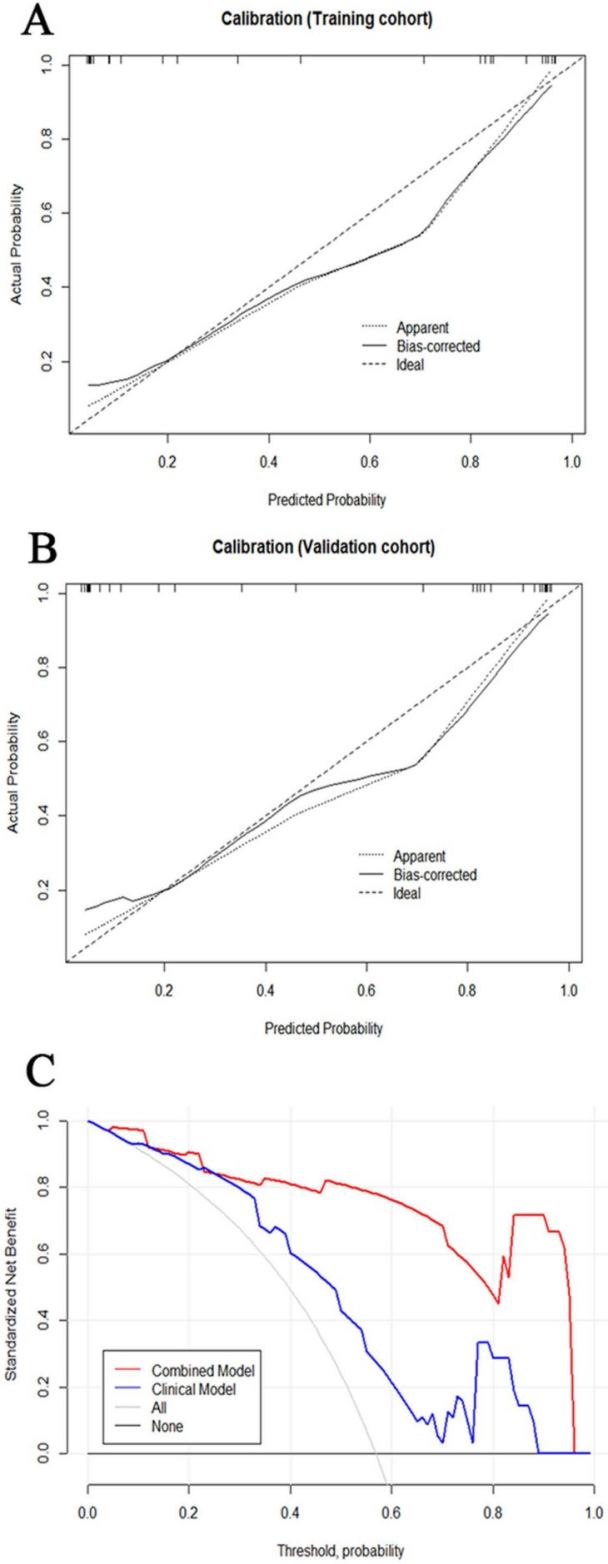
(A, B) Calibration curve of the nomogram in the training cohort and validation cohort. The curves reflect the consistency of the predicted result and the actual risk discrimination. Since the solid line is closed to the dotted line, the predictive power of the model was reliable. (C) Decision curve analysis of the radiomic nomogram demonstrated that when the threshold probability is between 0.06 and 0.95, the net benefit of using the radiomic nomogram to predict low-risk ECs is greater than treat-all or treat-none scheme.

The tumor size and nine retained radiomic signatures were identified as independent factors using multivariable logistic regression analysis. A nomogram derived from the combined model for predicting the probability of low-risk ECs is construsted (Fig. 4). The score of each factor were weighted by the hazard ratio. For each patient, a score was given for each predictive factor, and then a total score was acquired by adding up the score of all factors. Finally, corresponded predicted value of the total score was used to predict the risk level of the specific patient of EC. The predictive performance of aforementioned models are shown in Table 4 and ROC curves are presented in Fig. 5. The predictive efficacy of combined model is better than that of clinical model (Z=2.356, P=0.013) and radiomic model (Z=2.088, P=0.029), separatelly. The calibration curves of the nomogram are shown in Fig. 6A & B demonstrating good agreement in the training (p = 0.0.935) and validation (p = 0.638) cohorts. The nomogram showed a significant ability of risk straitification (Fig. 6C). When the threshold probability is between 0.06 and 0.95, the net benefit of using the radiomic nomogram to predict low-risk ECs is good. The NRI of was 0.28 (95% CI: 0.18-0.37), and the IDI was 0.031 (95% CI: 0.012-0.045), respectively.

## Discussion

In this research, a combined predictive model based on MR images and clinical parameters for preoperative risk stratifications in EC patients was established. It is excellent to predict low-risk ECs with an AUC of 0.889 in the validation cohort. The clinical features associating with the risk stratification were tumor size and DMI. Nevertheless, the clinical model based on these factors had limited performance. Radiomic features based on MR images were more sensitive than clinical features which exhibited higher specificity. The nomogram based on the combined model showed a better performance, with AUC of 0.955 (95% CI: 0.899-1) in the training and 0.889 (95% CI: 0.7-0.989) in the validation cohorts. Accordingly, we considered that the nomogram can be used to assist in predicting low-risk ECs non-invasively.

Several studies have explored the value of conventional MRI characteristics for preoperative risk stratification of EC 5, 7, 16-19. Lavaud et al. 20 found that tumor size reflects tumor grade, histologic type and lymphatic vascular invasion in EC, and a diameter of 24 mm should be used to preoperatively classify the high-intermediate or high-risk groups of stage 1 EC. Bourgioti et al. 5 also concluded that maximal tumor diameter can independently predict deep myometrial invasion on preoperative MRI. As these studies demonstrated, tumor size was a predictor of lymph node metastasis, deep myometrium invasion and tumor grade, thus it could be used as a diagnostic factor of risk stratification. In line with this, we find a tendency of low-risk tumors being smaller than high-risk tumors. The absence of DMI is also part of the definition of low-risk group, so the difference between these two groups is statistically significant. However, this outcome is contrary to that of Sahin et al. 18, who found that it was not statistically significant for tumor size, and tumor volume to predict tumor risk group. The clinical model, which was established by tumor size and DMI, yielded an AUC of 0.704 (95% CI 0.396-1) in the validation cohort. Our results indicated that tumor size in preoperative MR imaging may have the potential to preoperatively predict risk groups and thus contributes to optimizing therapy, and our radiomic texture model could further improve risk stratification.

The relationship between tumor texture characteristics and EC diagnosis and grading has been reported previously 11. Yang et al. 21 found that textural analysis based on T2-weighted MRI (DW-MRI) could discriminate different risk groups in rectal cancer accurately. We also chose T2-weighted images as the original data for texture analysis (TA) to evaluate EC stratification. A prospective cohort study showed that MRI-derived first-order texture parameters could provide a refined preoperative risk assessment in endometrial cancer 15. Yoshiko et al. 16 also evaluated the association among texture parameters and preoperative risk factors of EC, they found that the AUC was 0.83 (95% CI: 0.76, 0.89) for high-grade EC. In the present study, high-throughput parameters were extracted than previous studies and finally 9 statistically significant features were identified to build radiomic model. It is found that this radiomic model could discriminate between low-risk and intermediate-high risk EC groups; the AUC was 0.815 with a sensitivity and a specificity of 77.8% and 88.3% in the validation cohort.

Furthermore, we established a combined prediction model which integrating the tumor size and radiomic features. This combined model gained higher AUC and classification accuracy than that of the clinical model or radiomic model separately (P < 0.05). Thus, this combination has a better diagnostic performance than that of the prementioned models alone. Based on the combined model, a nomogram was established as a practical tool to demonstrate the risk group for each EC patient. Several studies have focused on the value of texture analysis and clinical parameters for preoperative risk stratification of EC 11-13, 22. A latest study by Yan et al. 13 indicated that MRI-based radiomics nomogram achieved a prominent predictive accuracy (AUC, 0.919 (95% CI: 0.879-0.960)) for the risk prediction in EC preoperatively. The large amount of data and multiple validation sets may be the reason for their excellent performance. In our study, the nomogram that established based on the clinical and radiomic features also showed a good diagnostic performance (AUC, 0.889 (0.7-0.989)), which is in consistent with previous study. In addition, decision curve analyses demonstrated that the nomogram in our study achieved good net benefit for EC risk stratification. The IDI and NRI showed that the clinical benefits of combined predictive model were improved comparing with the clinical or radiomic model alone, indicating that it could be an effective tool for clinical decision making.

There were several limitations in our study. Firstly, as a retrospective study, the MR images used was provided with various scanning parameters, so unnecessary confounding variability may be caused. Though normalization was employed during images analysis in this study, further standardization of the images should be investigated. Secondly, we employed only one sequence for texture analysis, which may potentially enable unnecessary confounders. Future studies should adopt multiparametric approaches to reduce the risk of biases from one sequence alone. Thirdly, large-scale data is necessary for the validation of radiomics represents. Fourthly, prognostic information was not incorporated into our models, more studies are necessary to explore how radiomic signatures can improve prognosis prediction in EC.

In conclusion, the combined model integrating the tumor size and radiomic signature had a robust predictive value for patients of low risk or intermediate-high risk EC. The nomogram based on this model a noninvasive and applicable tool for the clinical diagnosis and optimal decision-making of EC.

## Figures and Tables

**Table 1:** Patients' clinical characteristics and tumor pathological features

Characteristic	Data
Age (mean ± SD) (years)	57.8 ± 9.7
**Menopausal status**	
Premenopausal	13 (12.7%)
Postmenopausal	89 (87.3%)
**Histology**	
Endometrioid adenocarcinoma	84 (82.4%)
Serous Carcinoma	13 (12.7%)
Clear Cell Carcinoma	5 (4.9%)
**FIGO stage**	
Ia	79 (77.5%)
Ib	23 (22.5%)
**Tumor grade**	
Grade 1	65 (63.7%)
Grade 2	25 (24.5%)
Grade 3	12 (11.8%)
**Myometrial invasion**	
<50%	73 (71.6%)
≥50%	29 (28.4%)
**Lymphvascular invasion**	
Positive	80 (78.4%)
Negative	22 (21.6%)
**Risk stratification (stage I)**	
Low-risk group	69 (67.6%)
Intermediate-risk group	22 (21.6%)
High-risk group	11 (10.8%)

**Table 2:** Clinical and Conventional MRI features of the patients

MRI features	Low risk (n=69)	Intermediate-high risk (n=33)	*P*	Training cohort (n = 70)	Validation cohort (n = 32)	*P*
Age	57.7 ± 10.6	58.2 ± 10.1	0.82	57.1 ± 11.2	59.6 ± 8.3	0.25
**Menopausal status**			0.63			0.49
Premenopausal	9 (13%)	4 (12.9%)		10 (14.3%)	3 (9.4%)	
Postmenopausal	60 (87%)	29 (87.1%)		60 (85.7%)	29 (90.6%)	
Tumor Size (cm)	2.68 ± 1.12	4.08 ± 1.95	< 0.01	2.99 ± 1.55	3.43 ±1.58	0.19
**Tumor margin**			0.02			0.09
Well-defined	23 (33.3%)	4 (12.1%)		15 (21.4%)	12 (37.5%)	
Ill-defined	46 (66.7%)	29 (87.9%)		55 (78.6%)	20 (62.5%)	
**Signal on DWI**			0.06			0.31
Highintensity	62 (89.9%)	33(100%)		64 (91.4%)	31 (96.9%)	
Isointensity	7 (10.1%)	0		6 (8.6%)	1 (3.1%)	
**T1WI signal intensity**			0.15			0.86
Heterogeneous	3 (4.3%)	4 (12.1%)		5 (7.1%)	2 (6.2%)	
homogeneous	66 (95.7%)	29 (87.9%)		65 (92.9%)	30 (93.8%)	
**T2WI signal intensity**			0.49			0.75
Heterogeneous	61 (88.4%)	30 (90.9%)		62 (88.6%)	29 (90.6%)	
Homogeneous	8 (11.6%)	3 (9.1%)		8 (11.4%)	3 (9.4%)	
**Deep myometrial invasion**			< 0.01			0.97
Positive	0 (0%)	25 (75.8%)		26 (37.1%)	12 (37.5%)	
Negative	69(100%)	8 (24.2%)		44 (62.9%)	20 (62.5%)	

**Table 3:** Characteristics of selected radiomics features

Features	Coefficients	Mean	Standard deviation
LongRunHighGreyLevelEmphasis_AllDirection_offset5_SD	0.0318	9386217	16214286
RelativeDeviation	0.037	0.003	0.014
LongRunLowGreyLevelEmphasis_angle45_offset9	0.043	0.079	0.456
Correlation_angle0_offset9	0.076	0.166×10^-3^	0.778×10^-3^
InverseDifferenceMoment_angle135_offset3	0.136	0.048	0.019
Correlation_AllDirection_offset9	0.167	0.107×10^-3^	0.284×10^-3^
HighGreyLevelRunEmphasis_AllDirection_offset9_SD	0.344	33.079	61.608
skewness	0.347	0.401	0.703
Correlation_angle135_offset7	0.3575	0.007×10^-3^	0.004×10^-3^

**Table 4:** The predictive performance of the models

Models	Training cohort				Validation cohort					
	AUC (95%CI)	ACC	SPE	SEN	Cut-off	AUC (95%CI)	ACC	SPE	SEN	Cut-off
Clinical model (tumor size)	0.756 (0.65-0.86)	0.730	0.710	0.855	0.409	0.704 (0.396-1)	0.851	0.530	0.943	0.412
Radiomic model	0.946 (0.88-0.97)	0.892	0.965	0.810	0.760	0.815 (0.588-1)	0.8	0.833	0.768	0.673
Combined model	0.955 (0.899-1)	0.892	0.875	0.905	0.558	0.889 (0.7-0.989)	0.867	0.975	0.778	0.810

ACC: accuracy, SEN: sensitivity, SPE: specificity.

## References

[1] Brooks RA, Fleming GF, Lastra RR (2019). Current recommendations and recent progress in endometrial cancer. CA Cancer J Clin.

[2] Siegel RL, Miller KD, Jemal A (2020). Cancer statistics, 2020. CA Cancer J Clin.

[3] Colombo N, Creutzberg C, Amant F (2016). ESMO-ESGO-ESTRO Consensus Conference on Endometrial Cancer: Diagnosis, Treatment and Follow-up. Int J Gynecol Cancer.

[4] Eggemann H, Ignatov T, Kaiser K (2016). Survival advantage of lymphadenectomy in endometrial cancer. J Cancer Res Clin Oncol.

[5] Bourgioti C, Chatoupis K, Tzavara C (2016). Predictive ability of maximal tumor diameter on MRI for high-risk endometrial cancer. Abdominal radiology (New York).

[6] Canlorbe G, Bendifallah S, Laas E (2016). Tumor Size, an Additional Prognostic Factor to Include in Low-Risk Endometrial Cancer: Results of a French Multicenter Study. Ann Surg Oncol.

[7] Fasmer KE, Bjørnerud A, Ytre-Hauge S (2018). Preoperative quantitative dynamic contrast-enhanced MRI and diffusion-weighted imaging predict aggressive disease in endometrial cancer. Acta radiologica (Stockholm, Sweden: 1987).

[8] Liu J, Yuan F, Wang S (2019). The ability of ADC measurements in the assessment of patients with stage I endometrial carcinoma based on three risk categories. Acta radiologica (Stockholm, Sweden: 1987).

[9] Korkmaz V, Meydanli MM, Yalcin I (2017). Comparison of three different risk-stratification models for predicting lymph node involvement in endometrioid endometrial cancer clinically confined to the uterus. J Gynecol Oncol.

[10] Lambin P, Leijenaar RTH, Deist TM (2017). Radiomics: the bridge between medical imaging and personalized medicine. Nat Rev Clin Oncol.

[11] Xu X, Li H, Wang S (2019). Multiplanar MRI-Based Predictive Model for Preoperative Assessment of Lymph Node Metastasis in Endometrial Cancer. Front Oncol.

[12] Yan BC, Li Y, Ma FH (2020). Preoperative Assessment for High-Risk Endometrial Cancer by Developing an MRI- and Clinical-Based Radiomics Nomogram: A Multicenter Study. Journal of magnetic resonance imaging: JMRI.

[13] Yan BC, Li Y, Ma FH Radiologists with MRI-based radiomics aids to predict the pelvic lymph node metastasis in endometrial cancer: a multicenter study. Eur Radiol. 2020 Aug 4. Epub ahead of print.

[14] Bi WL, Hosny A, Schabath MB (2019). Artificial intelligence in cancer imaging: Clinical challenges and applications. CA Cancer J Clin.

[15] Liu Z, Wang S, Dong D (2019). The Applications of Radiomics in Precision Diagnosis and Treatment of Oncology: Opportunities and Challenges. Theranostics.

[16] Ueno Y, Forghani B, Forghani R (2017). Endometrial Carcinoma: MR Imaging-based Texture Model for Preoperative Risk Stratification-A Preliminary Analysis. Radiology.

[17] Body N, Lavoué V, De Kerdaniel O (2016). Are preoperative histology and MRI useful for classification of endometrial cancer risk?. BMC Cancer.

[18] Sahin H, Sarioglu FC, Bagci M (2018). Preoperative Magnetic Resonance Volumetry in Predicting Myometrial Invasion, Lymphovascular Space Invasion, and Tumor Grade: Is It Valuable in International Federation of Gynecology and Obstetrics Stage I Endometrial Cancer?. International journal of gynecological cancer: official journal of the International Gynecological Cancer Society.

[19] Vieillefosse S, Huchon C, Chamming's F (2018). Assessment of different pre and intra-operative strategies to predict the actual ESMO risk group and to establish the appropriate indication of lymphadenectomy in endometrial cancer. J Gynecol Obstet Hum Reprod.

[20] Lavaud P, Fedida B, Canlorbe G (2018). Preoperative MR imaging for ESMO-ESGO-ESTRO classification of endometrial cancer. Diagn Interv Imaging.

[21] Yang L, Liu D, Fang X (2019). Rectal cancer: can T2WI histogram of the primary tumor help predict the existence of lymph node metastasis?. European radiology.

[22] Xie H, Hu J, Zhang X (2019). Preliminary utilization of radiomics in differentiating uterine sarcoma from atypical leiomyoma: Comparison on diagnostic efficacy of MRI features and radiomic features. European journal of radiology.

